# Acute Necrotizing Encephalopathy in a Four-Year-Old Boy

**DOI:** 10.3390/diagnostics11030568

**Published:** 2021-03-22

**Authors:** Cheng-Hsien Tsai, Wei-Sheng Lin

**Affiliations:** 1 Department of Pediatrics, National Taiwan University Hospital Yunlin Branch, Yunlin 640, Taiwan; chtsa60@ntu.edu.tw; 2 Department of Pediatrics, Taipei Veterans General Hospital, Taipei 112, Taiwan; 3 Faculty of Medicine, National Yang-Ming University, Taipei 112, Taiwan

**Keywords:** acute necrotizing encephalopathy, thalamus, hydrocephalus

## Abstract

Acute necrotizing encephalopathy is a devastating clinico-radiological syndrome characterized by fulminant neurological deterioration after an antecedent febrile illness, as well as the imaging hallmark of bilateral thalamic involvement. Herein, we describe a 4-year-old boy with typical clinical and neuroimaging features of acute necrotizing encephalopathy. The bithalamic swelling led to a block of cerebrospinal fluid circulation at the foramen of Monro, thereby causing the mild dilatation of lateral ventricles. The periventricular areas could, therefore, have been potentially affected by the acute necrotizing encephalopathy per se and/or transependymal edema secondary to obstructive hydrocephalus. The information from diffusion imaging allows for differentiation between these two pathophysiological processes.

A previously healthy 4-year-old boy presented with fever for one day, accompanied by vomiting and diarrhea. He was admitted for supportive care. However, consciousness disturbance and seizure developed on the day of hospitalization. A diagnostic lumbar puncture was performed, revealing elevated leukocyte count (30 cells per µL), erythrocyte count (190 cells per µL), and protein level (212 mg/dL) in cerebrospinal fluid. Magnetic resonance imaging of the brain on the next day revealed the mild dilatation of lateral ventricles (Figure 1a,b), as well as signal changes on T2-weighted sequences (Figure 1a–d) and restricted water diffusion (Figure 1e,f) in the bilateral thalami, bilateral periventricular regions, brainstem, and cerebellum. He was diagnosed with acute necrotizing encephalopathy (ANE) with obstructive hydrocephalus due to thalamic swelling (arrows). High-dose methylprednisolone was instituted, and external ventricular drainage was performed. The intraoperative measurement of intracranial pressure was 17 cm H_2_O.

ANE is a distinct clinico-radiological syndrome characterized by fulminant neurological deterioration following a febrile illness, as well as bilateral thalamic involvement on neuroimaging. The radiological picture of bilateral diffuse thalamic lesions with mass effect, as illustrated in this case, may occasionally mimic bithalamic glioma [1], Leigh syndrome [2], thrombosis of internal cerebral veins and other deep sinovenous structures [3], or flavivirus encephalitis [4]. Nonetheless, these diagnoses could usually be differentiated from ANE on the basis of clinical manifestations and ancillary laboratory information. On the other hand, the periventricular hyperintense signals on T2-weighted sequences (Figure 1a,b) may bear a superficial resemblance to the transependymal resorption of cerebrospinal fluid secondary to obstructive hydrocephalus. However, diffusion is usually increased in the latter [5], while it was restricted in these areas in our case (e.g., compare Figure 1b,e), suggesting the presence of cytotoxic edema. Admittedly, the neuroimaging features of ANE are likely highly dynamic and ever-changing [6,7]. Further research is needed to clarify how the diffusion characteristics evolve along the disease course.

ANE has been reported across different age groups and geographic regions, while it is most often seen in young children in East Asia [8]. Both genetic predisposition and an infectious trigger have been implicated in the etiopathogenesis [9,10]. The mainstay of treatment is immunomodulatory medications, while external ventricular drainage or other surgical measures were occasionally needed or contemplated for acute hydrocephalus with intracranial hypertension [6,11]. Although ANE is rare, it could complicate the course of a variety of common infections, from influenza to COVID-19 [7,9,12]. No pathogen was identified in our patient despite extensive evaluation. He survived with profound mental and motor deficits and was bedridden at 1-year follow-up.

## Figures and Tables

**Figure 1:**
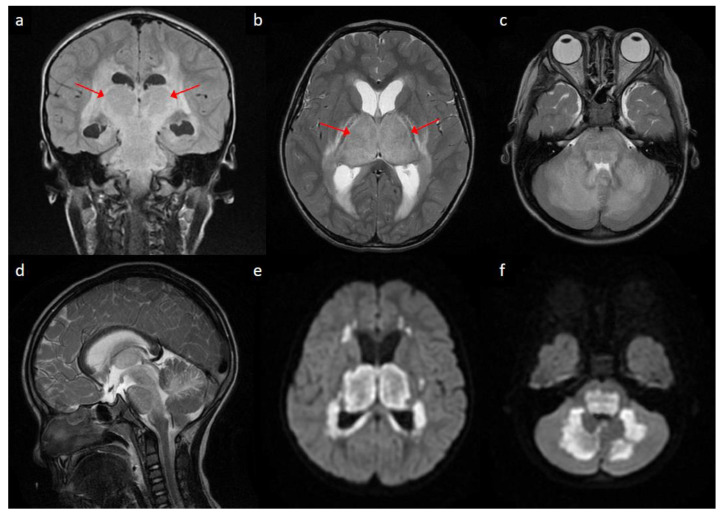
Brain magnetic resonance imaging of the patient: (**a**) coronal view of T2 fluid-attenuated inversion recovery sequence showing hyperintense signals in bilateral thalami (arrows) and periventricular regions; (**b**,**c**) Axial view of T2-weighted sequences at the level of basal ganglia/thalami (arrows) and pons/cerebellum, respectively, showing relatively symmetric hyperintense signals in bilateral thalamic, periventricular, pontine and cerebellar regions; (**d**) sagittal view of T2-weighted sequence showing hyperintense signal changes in brainstem and cerebellum; (**e**,**f**) axial view of diffusion-weighted imaging at the levels corresponding to (**b**,**c**), respectively, showing bright signals in bilateral thalamic, periventricular, pontine and cerebellar regions.
